# Beer as a Rich Source of Fluoride Delivered into the Body

**DOI:** 10.1007/s12011-016-0888-8

**Published:** 2016-11-04

**Authors:** D. Styburski, I. Baranowska-Bosiacka, M. Goschorska, D. Chlubek, I. Gutowska

**Affiliations:** 1 Department of Biochemistry and Human Nutrition, Pomeranian Medical University in Szczecin, Broniewskiego 24 street, 71-460 Szczecin, Poland; 2 Department of Biochemistry, Pomeranian Medical University, Powstańców Wlkp. av. 72, 70-111 Szczecin, Poland

**Keywords:** Beer, Fluoride content, Fluoride intake

## Abstract

Fluoride is an element which in the minimum amount is necessary for the proper construction of the teeth and bones. But on the other hand, it increases the synthesis of reactive oxygen species, inflammatory mediators, and impairs the action of enzymes. Beer is the most popular alcoholic beverage in the world. Due to its prevalence and volume of consumption, it should be considered as a potential source of F- and taken into account in designing a balanced diet. Therefore, the aim of this study was to analyze beer samples in terms of F- levels. The concentrations of fluoride were examined using ion-selective electrode Thermo Scientific Orion and statistical analysis was based on two-way ANOVA and *t* test. When compared to imported beers, Polish beers were characterized by the lowest mean F- concentration (0.089 ppm). The highest mean F- concentrations were recorded in beers from Thailand (0.260 ppm), Italy (0.238 ppm), Mexico (0.210 ppm), and China (0.203 ppm). Our study shows that beer is a significant source of fluoride for humans, which is mainly associated with the quality of the water used in beer production.

## Introduction

Fluoride (F-) is a common component of inanimate and animate matter [1]. Considerable amounts can be found in water, air, and a variety of foodstuffs, which results in considerable exposure to living organisms, including humans [2–5]. Along with its high accumulative properties, fluoride’s high reactivity may affect many biochemical processes; this leads to the synthesis of reactive oxygen species, inflammatory mediators, and disruption of enzyme activity [6]. Excess F- is a major cause of dental and skeletal fluorosis [7, 8] and the increasing number of studies link excess concentrations of F- with disturbances in the functioning of many organs, the development of atherosclerosis [9, 10], and neurodegenerative diseases [11–13].

Beer is the most popular alcoholic beverage in the world and the third most popular drink after water and tea [14]. Due to its prevalence and volume of consumption, it should be considered as a potential source of F- and taken into account in designing a balanced diet [15]. Because of the scarcity of publications concerning the concentrations of F- in beers, as well as the fact that beer is a very good endogenous source of F-, it seems reasonable to examine the content of F- ions in various types of beer as a potential source of F- for humans [16, 17]. Therefore, the aim of this study was to analyze beer samples in terms of F- levels. Our research was to determine whether beer is a significant source of F- for humans and find potential differences in F- levels between beer types and shops where they were purchased, as well as the countries of origin.

## Material and Methods

### Samples

The study involved a total of 69 samples of beers produced in Poland as well as those imported into Poland from Asia, South America, and various European countries. The samples were collected in 2014 and 2015 in shops in the Zachodniopomorskie and Wielkopolskie voivodships. Twenty milliliters of beer was poured from a can or bottle into a plastic tube, labeled, and frozen at −20 °C until the determination of F- levels.

### Determination of Fluoride Content in Collected Samples

Fluoride concentrations in individual samples were measured by potentiometric method with a fluoride ion-selective electrode (Orion 9409 BN, Thermo Scientific, USA). Carbonated drinks were degassed before the measurements [18, 19]. Next, 1 mL of sample was transferred to a plastic tube and then 1 mL of TISAB II was added to this solution. After mixing, the potential difference of each sample was measured for 10 min: 5 min before the addition of the appropriate standard and 5 min after the addition. According to the work of Łukomska et al. [4], the fluoride content in samples was calculated based on the difference of potentials measured in each sample and the concentration of the added standard. The correctness of the analytical procedure was controlled by determining the concentration of F- in NaF solutions with known concentrations: 0.1, 1, and 10 mg/kg (Orion Company, USA).

### Statistical Analysis

Statistical analysis used R Project software. Parametrics of data was determined with the Anderson-Darling test, and the homogeneity of variance with Bartlett’s test. The data in the study was parametric, which allowed the use of parametric tests in the study. Then, analysis was performed with two-way ANOVA and *t* test. The level of significance was *p* ≤ 0.05.

## Results

**Fig. 1:**
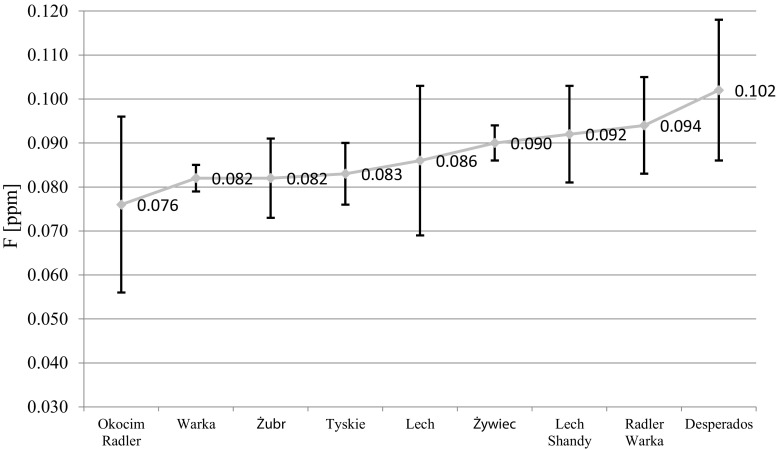
Fluoride mean concentration and SD in various brands of beers produced in Poland

In analyzing the Polish-made beer samples, the lowest F- concentration was observed in Okocim Radler at 0.076 ppm. The median F- level for the Polish-made beers was 0.084 ppm, while mean F- was 0.089 ppm. Within the entire group of Polish-made beers, the highest F- level was observed in Desperados beer (0.102 ppm) (Fig. 1). However, two-way ANOVA analysis showed no statistically significant differences in this group (*p* > 0.05).

**Fig. 2:**
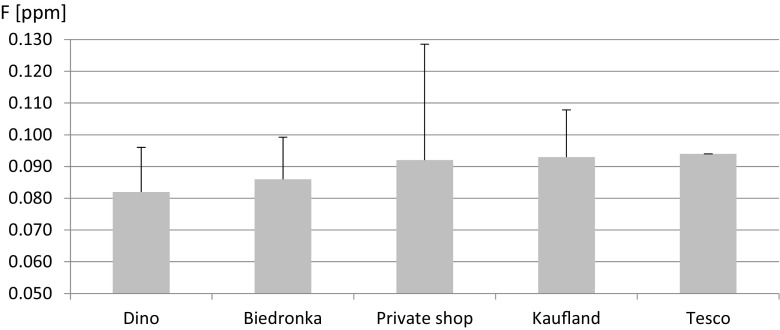
Fluoride mean concentration and SD in beers from different shops

In the second part of the study, we analyzed F- levels in beers by place of purchase. The lowest mean F- levels were observed in beers from Dino (0.082 ppm) and Biedronka (0.086 ppm) chains, while higher values were observed in those from Tesco (0.094 ppm), Kaufland (0.093 ppm), and private stores (0.092 ppm) (Fig. 2). However, we observed no statistically significant correlations between F- levels and the place of purchase (*p* > 0.05).

**Fig. 3:**
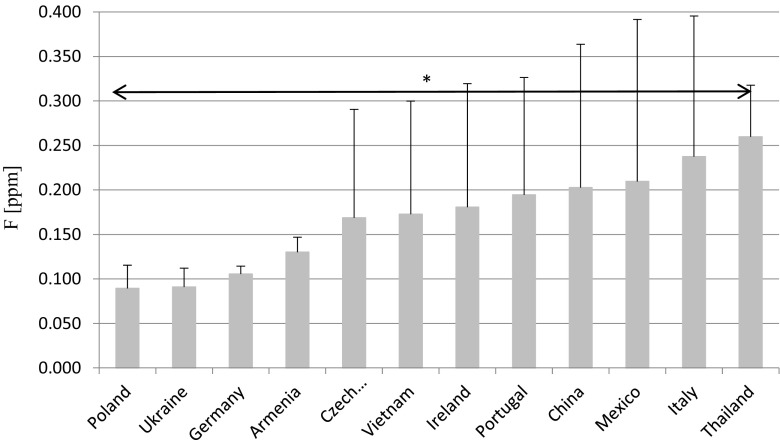
Fluoride mean concentration and SD in beers from different countries. *Statistical significant differences *p* ≤ 0.05

Comparing F- levels between Polish and imported beers, we observed distinct differences depending on the country of origin. When compared to imported beers, Polish beers were characterized by the lowest mean F- concentration (0.089 ppm). Low mean F- levels were also observed in the Ukrainian (0.091 ppm), German (0.106 ppm), and Armenian (0.130 ppm) beers. Much higher mean F- concentrations were recorded in beers from Thailand (0.260 ppm), Italy (0.238 ppm), Mexico (0.210 ppm), and China (0.203 ppm) (Fig. 3). Beers from the Czech Republic, Vietnam, Ireland, Portugal, China, Mexico, and Italy had much higher standard deviations than the Polish, Ukrainian, German, and Armenian beers, which may indicate a wide variation in F- content in the waters of those countries. Therefore, statistical analysis demonstrated statistically significant differences in F- levels only between Polish and Thai beers (*p* < 0.05).

## Discussion

Water is an essential raw material in beer production. To a large extent, its quality and chemical composition determines the quality of the final product. The chemical composition of water is crucial for the specific taste and therapeutic properties of beer [20]. While the absorption of F- in low concentrations found in a variety of sources helps protect teeth against decay, an excess can lead to fluorosis [4], commonly observed in areas endemically rich in F-, e.g., China, Ethiopia, and Kenya [17, 15, 23]. As the anticaries properties of F- and the occurrence of adverse effects in the body depend on the administered dose, it is important to find and assess potential sources of F- in order to determine the optimal concentrations for the anticaries effect and minimize the risk of fluorosis [16]. The US-recommended Adequate Intake Levels (AI) for F- are 3–4 mg/day and the Upper Intake Level (UL) is 10 mg/day for adults (Dietary Reference Intakes recommended by the National Academy of Sciences Food and Nutrition Board, USA) [24]; in Poland, RDA for F- is 3–4 mg/day for adults (Food and Nutrition Institute in Warsaw). Importantly, the recommended daily intake of F- varies depending on gender and age [18]. In addition, F- levels in water samples originating from a given area can vary considerably, as shown by research on F- in drinking water in Chile [16]. In a study on Lake Victoria in Africa with water F- levels exceeding the standards set by the World Health Organization and increasing the risk of fluoride-dependent diseases, groundwater levels of F- decreased in proportion to the distance from the lake. The author of that paper suggests that considerable amounts of fluorine compounds may come from nonanthropogenic sources, e.g., the areas significant in volcanic activity, which shows that even in poorly industrialized areas, F- can become a serious problem and pose a danger to the health of humans and animals [21].

The level of F- contained in alcohol is mainly related to the chemical composition of the water, and therefore beer or wine—alcoholic beverages with the lowest amount of alcohol—tend to have the highest F- levels [17]. Research conducted in the Ethiopian Rift Valley showed significantly higher levels of F- in regional beers and meads than in the drinking water, most likely caused by cooking fermented grain in wells containing water with high F- levels. In addition, grain grown in regions with high soil F- levels may also accumulate this element. Observations have confirmed that drinkers of Ethiopian alcoholic beverages had a significantly higher risk of fluorosis than nondrinkers, confirming the role of general local Ethiopian alcohol as a source of F- and the impact of water quality on F- levels in the beers [15].

It is believed that the level of F- in beers can affect the process of mashing, responsible for the breakdown of starch contained in grains fermented by yeast. Beer malts may have F- levels as high as 12 ppm. However, some research show that F- remains in draff once it is separated from the wort, which does not increase this element content in beers [14, 22]. Another paper, comparing mineral water and tap water used in the production of wort, demonstrated that F- in the mineral water reduces its acidity, which is significant as increased pH decreases protein degradation in wort and inhibits proteolytic enzymes, for example, those responsible for starch breakdown. In turn, these processes are crucial for the quality and taste of beer, frothing, and shelf life. In addition, higher pH enhances the extraction of some undesirable compounds to wort, e.g., polyphenols, detrimental to its quality and color [20]. The concentration of F- in beers may also depend on the place of production. For example, Carlsberg and Heineken beers made in the UK have lower F- levels, the same with beers brewed in Denmark and the Netherlands. Differences in the level of F- also occur between bottled and canned beers, as F- can be absorbed by glass [23].

In the UK, the daily recommended dose of F- is 1.82 mg/day, of which a significant percentage comes from teas and other beverages [4, 23]. This leaves a narrow margin of tolerance for other sources of F- and makes some groups of people, e.g., alcoholics, exposed to especially high doses of F-. As alcoholics may intake more than 50 units of alcohol a week [23] and the Institute of Food and Nutrition defines one dose of beer to be 330 ml [25], alcoholics may drink more than seven doses of beer a day (2.3 l a day). A poll survey on alcohol drinking in Poland shows that 84% of Poles consume alcohol, most of whom are men, while at the European level, 76% of the population consumes alcohol. Polish society in 2012 consumed an average of 9.25 l of alcohol per person, and beer was the most popular choice for alcohol consumption (87.6%) [17].

This study analyzed the concentration of F- in Polish and imported beers. F- levels in Polish beers were similar between the analyzed brands and lower when compared to that of the imported beers. The mean concentration of F- in Polish beers was 0.089 ppm, (0.076 to 0.102 ppm). These results are much lower when compared with the results of a paper on F- levels in beer in the UK, which ranged from 0.15 to 0.86 ppm [22], as well as in a paper on wines from the Canary Islands, where mean F- content amounted to 0.15 ppm. The apparent relation between the country of origin and F- level may be due, for example, to the geographical location and the degree of volcanic activity in the country [4]. Substantial amounts of F- produced by volcanic eruptions may accumulate in plants and enter the water used in beer production [4]. It is believed that this is the reason for high F- levels in Thai, Chinese, Mexican, and Italian beers, much higher compared to those in Polish, German, and Ukrainian beers. Another explanation for the higher content of this element in beers may be the use of artificially fluoridated water [17]. Beers from Mexico, Italy, China, and Vietnam were characterized by large standard deviations. It can be assumed that they are mainly caused by differences in natural F- levels in the waters in different areas of the country.

The results of the aforementioned scientific studies on F- concentrations in alcoholic beverages and water were designed to investigate the effects of drinking alcohol on the risk of clinical symptoms of fluorosis [2, 15, 21, 23]. New research shows that the negative effects of F- can occur before any external symptoms of the disease. Chronic exposure to low F- levels can significantly increase the synthesis of ROS and pro-inflammatory mediators in the body, processes responsible for the development of a number of inflammatory diseases [5]. A number of studies analyzed by Peckham and Awofeso [1] indicate that there is no safe level of fluoride for humans, and the universality and accessibility of this element in nature should result in measures aiming to reduce its consumption worldwide [1]. Many countries, Germany, the Netherlands, Sweden, and Switzerland, from fear of the consequences of the impact of excessive F- on the human body, have stopped water fluoridation. Currently, only 350 million of the world’s population drink fluoridated water, of which 200 million are in the USA. Although F- prevents the development of tooth decay, new reports suggest the need for a reduction in the intake of F- and prevention of tooth decay without the use of F- [1].

## Conclusions

Based on the studies, it can be concluded that beer is a significant source of fluoride ions to man. Compared with imported beers, beers produced in Poland had the lowest levels of fluorine. The study also noted significant differences in the concentration of fluoride in relation to the country in which a brand of beer is produced. These differences may be related to the water quality in the country and the region.
